# Meta-Analysis of the Effect of Honghua Injection in the Treatment of Coronary Heart Disease Angina Pectoris

**DOI:** 10.1155/2022/4537043

**Published:** 2022-05-25

**Authors:** Jiani Zhai, Zhaochen Ji, Xinyao Jin, Xuechen Du, Lujia Cao, Wenke Zheng

**Affiliations:** Tianjin University of Traditional Chinese Medicine, Tianjin 301600, China

## Abstract

**Objective:**

To evaluate the effectiveness of honghua injection combined with western medicine in the treatment of coronary heart disease angina pectoris.

**Methods:**

Computer extensively searched PubMed, Cochrane Library, Embase, China Biomedical Archives (SinoMed), China Knowledge Network (CNKI), Chinese Journal Full-text Database (VIP), Wanfang Data Knowledge Service Platform (Wanfang), and collected randomized controlled trials (RCTs) of honghua injection combined with western medicine in the treatment of coronary heart disease angina pectoris. Use Review Manager5.3 software for meta-analysis.

**Results:**

21 RCTs were included, involving 1894 participants. Meta-analysis shows that honghua injection combined with western medicine can significantly improve the clinical efficacy (OR = 4.03, 95% CI[2.96,5.49]), electrocardiographic efficacy (OR = 3.39, 95%CI[2.44, 4.70]), can significantly reduce total cholesterol (TC) levels (MD = −0.39, 95% CI[−0.47, −0.31]), triacylglycerol (TG) levels (MD = −0.45, 95% CI[−0.51, −0.39]), increase high-density lipoprotein cholesterol (HDL-C) levels (MD = 0.29,95%CI[0.26,0.32]), reduce low-density lipoprotein Cholesterol (LDL-C) levels (MD = −0.59, 95%CI[−0.79, −0.38]). Five articles reported adverse reactions.

**Conclusion:**

Honghua injection combined with western medicine is more effective than western medicine alone in patients with coronary heart disease angina pectoris. More multicenter, large sample, high-quality RCTs are needed to provide evidence.

## 1. Introduction

According to a survey by the World Health Organization (WHO), one cause of global lifespan reduction is coronary atherosclerotic heart disease (CHD), referred to as coronary heart disease [1]. With economic developing, people's living habits have changed and the population is aging, the risk factors of coronary heart disease are becoming increasingly prominent. According to estimates, as of 2018, there were about 11 million patients with coronary heart disease in China [2], and the mortality rate continues to rise, becoming a major disease threatening human health. Epidemiological studies have shown that the overall prevalence of coronary heart disease is on the rise, the patient population tends to be younger, and the number of acute attacks is increasing year by year [3]. Coronary heart disease has many complications and dangerous diseases. If it is acute, it can develop into myocardial infarction, and in severe cases, it can cause sudden death. Therefore, it is particularly critical to study its clinically effective treatment.

In the field of modern medicine, Coronary heart disease is usually caused by a single risk factor called “dyslipidemia,” and the main factors that cause dyslipidemia are genetics, lifestyle, certain diseases (such as thyroid dysfunction and kidney disease) and medications [4].he treatment of coronary heart disease often uses antiplatelet drugs, calcium antagonists, and other drugs, and symptomatic treatments such as anticoagulation, lipid regulation, blood pressure reduction, and vasodilation can significantly alleviate the clinical symptoms of patients. The effect of functional improvement is limited, and adverse drug reactions are prone to occur [5]. Traditional Chinese medicine believes that the pathogenesis of coronary heart disease is based on the right deficiency, and it is a process of intermixing with Yin and cold, blood stasis, qi stagnation, phlegm obstruction and other evils [6]. Clinical observations have shown that, compared with simple chemical treatment, using the appropriate treatment plan of traditional Chinese medicine preparations combined with conventional chemical drugs has a significant effect, and it has a better effect in improving the quality of life of patients and reducing side effects [7].

Honghua injection is an injection made by processing and extracting honghua. It is a clear liquid from yellowish red to reddish brown. At present, it has been widely used in various diseases, such as hypertension, coronary heart disease, pulmonary heart disease, cerebral infarction, diabetes, chronic renal failure, and primary dysmenorrhea [8]. Relevant studies have shown that honghua injection has the effects of improving blood concentration, anticoagulation, and improving microcirculation [9], and the pharmacological effects of honghua for promoting blood circulation, clearing menstruation, removing blood stasis and relieving pain are the expansion of blood vessels and anti-myocardial ischemia, lowering blood lipids, exciting the uterus, anticoagulation, anti-thrombosis, and other effects [10, 11].

Relevant clinical trials have initially shown that honghua injection combined with western medicine can improve the treatment effect of coronary heart disease angina pectoris. However, it has not been systematically evaluated in the world. In this study, methods such as meta-analysis were used to systematically evaluate the effectiveness of honghua injection combined with western medicine in the treatment of coronary heart disease angina pectoris, which is convenient to guide clinical use.

## 2. Materials and Methods

### 2.1. Literature Search

Computer search of PubMed, Cochrane Library, Embase, China Biomedical Archives (SinoMed), China Knowledge Network (CNKI), Chinese Journal Full-text Database (VIP), Wanfang Data Knowledge Service Platform (Wanfang) database for the RCT literature of honghua injection in the treatment of coronary heart disease angina pectoris, the search time is from the establishment of the database to October 2021. The Chinese search terms are “coronary atherosclerotic heart disease,” “coronary heart disease,” “coronary syndrome,” “angina pectoris,” “honghua injection,” “safflower injection” and “random.” The English search terms are “coronary disease,” “coronary artery disease,” “random,” “Honghua injection,” “Honghua injection” and so on.

### 2.2. Inclusion Criteria

(1) The type of research is RCT. (2) The subjects of the study are patients who meet the domestic and foreign consensus criteria for diagnosing coronary heart disease angina pectoris, regardless of age and gender. (3) The control group is treated with conventional western medicine, and the test group is treated with honghua injection on the basis of the control group. (4) The language is Chinese or English.

### 2.3. Exclusion Criteria

(1) Documents published repeatedly. (2) Documents for which data cannot be extracted. (3) In addition to honghua injection and conventional western medicine, the treatment group also used other traditional Chinese medicine injections. (4) The treatment group used honghua extract and other similar products.

### 2.4. Outcome Indicators

1. Clinical efficacy. It meets the “Criteria for Evaluating the Curative Effect of Coronary Heart Disease Angina Pectoris and ECG.” Significant effect: After treatment, the patient's ECG returned to normal or almost normal or reached the standard of marked effect, or the symptoms of angina pectoris disappeared or the number of angina pectoris was reduced by more than 80%, nitroglycerin consumption was reduced by more than 80%, or the main clinical symptoms improved within 10 days or the chest pain symptoms disappeared or Reduced by 90%; Effective: After treatment, the patient's angina pectoris symptoms were significantly relieved, the number of angina pectoris was reduced by more than half, nitroglycerin consumption was reduced by 50% to 80%, the ECG improvement reached the effective standard or the main clinical symptoms improved within 10 to 20 days, or chest pain symptoms Improved by more than 50%; ineffective: After treatment, the patient's angina pectoris symptoms are not improved or even worsened, the ECG is the same as before treatment or worse than before treatment, or the number of angina pectoris episodes, nitroglycerin consumption is reduced by less than 50%, or chest pain symptoms are not improved.
2. Efficacy of ECG. It meets the “Criteria for Evaluating the Efficacy of Coronary Heart Disease Angina Pectoris and ECG.” Markedly effective: ST-segment after treatment, T wave returned to normal or roughly normal range; effective: ST-segment of ECG after treatment increased by more than 0.05 mV compared to before treatment but did not reach the normal level, the inverted T wave changes in the main lead became shallow or changed two-way; ineffective: After treatment, the ECG is similar to that before treatment or the ST segment is lower than before treatment by more than 0.05 mV, and the T wave is deepened or not improved.
3. Blood lipid indicators [including total cholesterol (TC), triacylglycerol (TG), high-density lipoprotein cholesterol (HDL-C), low-density lipoprotein cholesterol (HDL-C)].
4. Adverse reactions.

### 2.5. Data Extraction

Two researchers independently screened the literature, extracted data, and cross-checked according to preset inclusion and exclusion criteria. If there is any objection, the third researcher will discuss and resolve it through negotiation. Establish an Excel data extraction table, which mainly includes: the source of the included literature (name of the first author, year of publication), sample size, general information of participants (such as gender, age, etc.), intervention measures, treatment process and outcome indicators.

### 2.6. Bias Risk Assessment

Use the Cochrane Risk of Bias Tool to assess the risk of bias for each study. The tool considers the following factors: (a) the random sequence generation method used; (b) the allocation concealment method used; (c) the blinding method used (participants and personnel); (d) the blinding method used (result evaluator); (e) incomplete result reporting; (f) selective reporting of research results and (g) other possible sources of bias. Each entry is judged as low risk, high risk, or unclear risk of bias.

### 2.7. Statistical Analysis

RevMan 5.3 software was used for statistical analysis of the included literature research data, and the count data used risk ratio (RR) or odds ratio (OR), continuous variable data was expressed as mean difference (MD), and 95% CI indicated that the difference was statistically significant. If *I*^2^ ≦ 50%, it indicates that the heterogeneity is small, and the fixed effects model (FEM) is used; If *I*^2^ > 50%, it indicates that the heterogeneity is large, first determine the reason for the heterogeneit, conduct a sensitivity analysis to eliminate the possible source of the heterogeneity, if the heterogeneity is still large, then use the random effects model (REM). Or use subgroup analysis for further analysis.

## 3. Results

### 3.1. Literature Search Results

**Figure 1:**
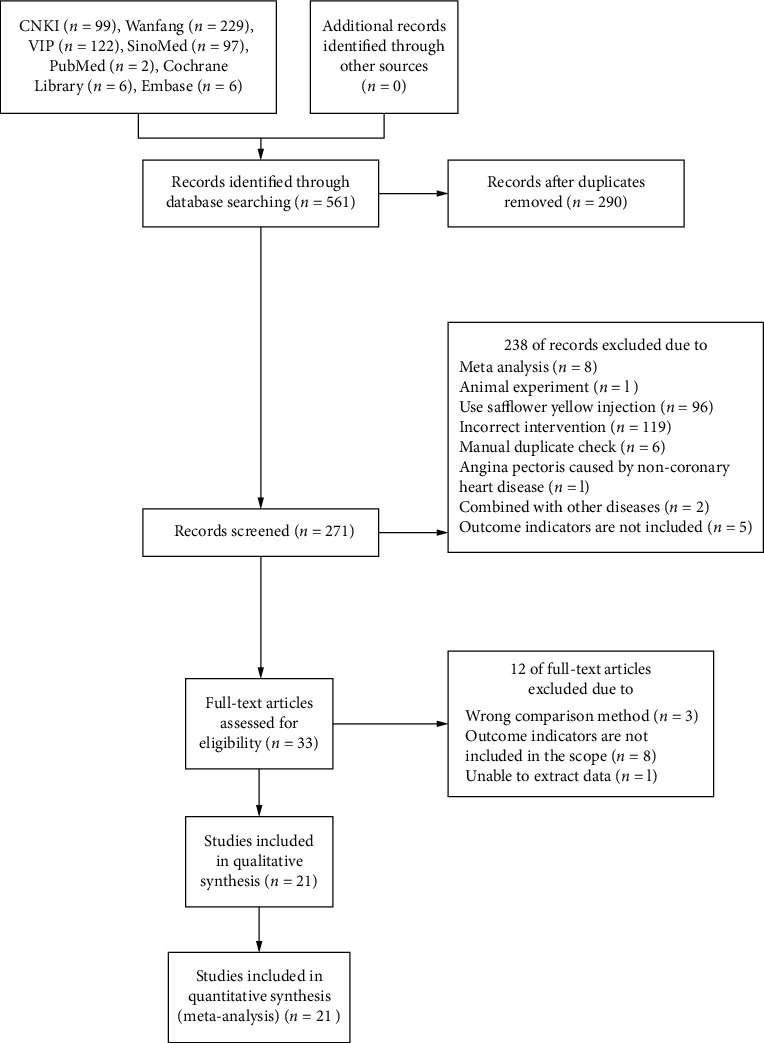
Flowchart of the literature retrieval.

As shown in Figure 1, 561 documents were initially retrieved, of which 290 studies were eliminated as duplicates. After screening by title, abstract, and full text, a total of 21 studies were included in this meta-analysis (Figure 1).

### 3.2. Incorporation of the Basic Features of the Literature

All included studies were published in China, and a total of 1894 patients diagnosed with coronary heart disease and angina pectoris were included, including 969 in the treatment group and 925 in the control group. 17 outcome index studies reported clinical efficacy [12–28], 11 studies reported ECG efficacy [14, 17, 19, 21–25, 27–29], 4 studies reported TC levels [16, 30–32], 4 studies reported TG levels [16, 30–32], 4 studies reported HDL-C levels [16, 30–32], 4 studies reported LDL-C levels [16, 30–32], 5 studies reported adverse reactions or events [12, 17, 23, 27, 28]. The control group was treated with western medicine, and the experimental group was treated with honghua injection combined with the control group. The course of treatment was 14–28 days. The characteristics of all included RCTs are summarized in Table 1.

### 3.3. Risk of Bias in the Included Literature

**Figure 2:**
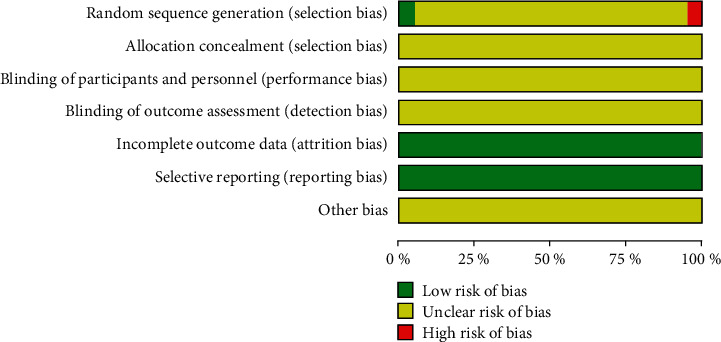
Risk and bias graph.

Nineteen studies [13–22, 29, 30, 32] only described “random grouping” and were considered unclear risk, and 1 study was included in the group order odd and even numbers were randomly divided into treatment group and control group, which were considered high risk. One study was divided into treatment group and control group according to the random number table, which was considered low risk. None of the included studies described allocation concealment and blinding, so they were assessed as unclear risks. The included studies reported that the outcome data was complete and there was no selective reporting, so the reporting bias was evaluated as low risk. None of the included trials described whether there were other potential biases, so the risk was determined to be unclear. The results of the risk of bias assessment are shown in Figure 2.

### 3.4. Research Results

#### 3.4.1. Clinical Efficacy

**Figure 3:**
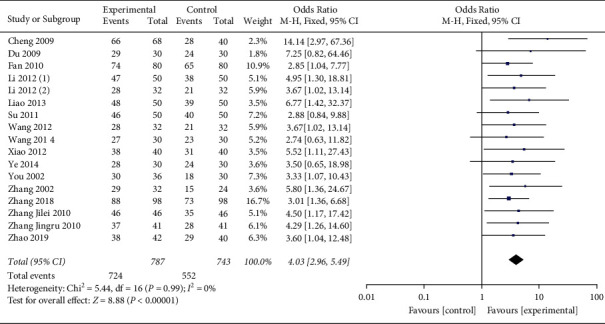
Forest plots of clinical efficacy.

Meta-analysis results showed that there was no heterogeneity among the 17 studies (*P*=0.99 9, *I*^2^ = 0%), so the fixed effects model was used, and the difference was statistically significant (OR = 4.03, 95%CI[2.96, 5.49], *P* < 0.00001), indicating that the clinical effect of honghua injection combined with western medicine in the treatment of coronary heart disease angina pectoris is better than that of the western medicine treatment group (Figure 3).

#### 3.4.2. Efficacy of ECG

**Figure 4:**
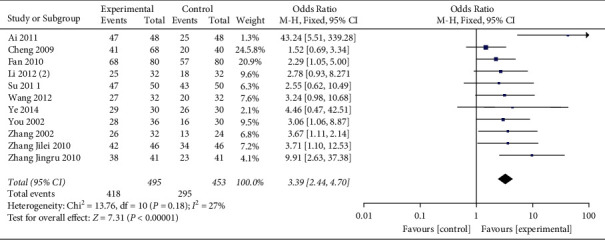
Forest plots of ECG efficiency.

Eleven studies have reported the efficacy of ECG.After the heterogeneity test, the heterogeneity among the studies were small (*I*^2^ = 27%, (*P*=0.18), so a fixed-effect model was used. The results showed that honghua injection combined with western medicine was better than the control group in improving the efficacy of ECG, and the difference was statistically significant (OR = 3.39, 95%CI [2.44, 4.70], *P* < 0.00001) (Figure 4).

#### 3.4.3. TC

**Figure 5:**
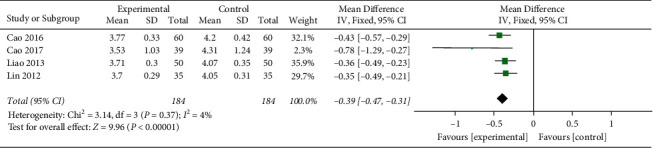
Forest plots of TC levels.

Four studies reported TC.After the heterogeneity test, the heterogeneity among the studies is small (*I*^2^ = 4%, (*P*=0.37), so the fixed effects model was used, and the difference was statistically significant (MD = -0.39, 95%CI[−0.47, −0.31], *P* < 0.00001) indicates that the TC levels of honghua injection combined with western medicine in the treatment of coronary heart disease angina pectoris is better than that in the western medicine treatment group (Figure 5).

#### 3.4.4. TG

**Figure 6:**
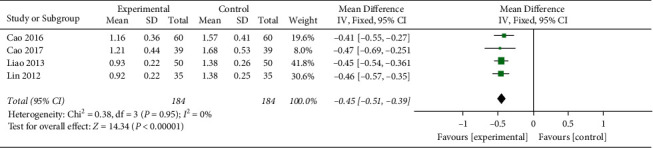
Forest plots of TG levels.

Four studies reported TG.After the heterogeneity test, there was no heterogeneity between the studies (*I*^2^ = 0%, (*P*=0.95), so the fixed effects model was used, and the difference was statistically significant (MD = −0.45, 95%CI[−0.51, −0.39], *P* < 0.00001) indicates that the TG levels of honghua injection combined with western medicine in the treatment of coronary heart disease angina pectoris is better than that in the western medicine treatment group (Figure 6).

#### 3.4.5. HDL-C

**Figure 7:**
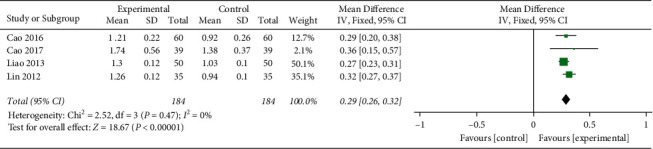
Forest plots of HDL-C levels.

4 studies reported HDL-C.After the heterogeneity test, there was no heterogeneity between the studies (*I*^2^ = 0%, (*P*=0.47), so the fixed effects model was used, the difference was statistically significant (MD = 0.29,95% CI [0.26, 0.32], *P* < 0.00001), indicating that the HDL-C levels of honghua injection combined with western medicine is better than that of the western medicine treatment group (Figure 7).

#### 3.4.6. LDL-C

**Figure 8:**
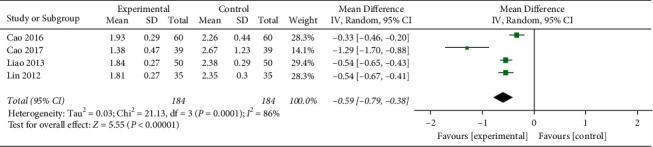
Forest plots of LDL-C levels.

4 studies reported LDL-C.After the heterogeneity test, the heterogeneity between the studies was relatively large (*I*^2^ = 86%, *P* < 0.0001). The sensitivity was analyzed, Excluding the literature one by one for heterogeneity test, the results show that the heterogeneity was still large, so the random effects model was used, and the difference was statistically significant (MD = −0.59, 95%CI[−0.79, −0.38], *P* < 0.00001) (Figure 8).

#### 3.4.7. Adverse Reactions

A total of 5 studies [12, 17, 23, 27, 28] reported adverse reactions. 2 studies [25, 29] reported no adverse reactions, 14 studies [13–16, 18–22, 24, 26, 30–32] did not mention adverse reactions (Table 2).

### 3.5. Publication Bias

**Figure 9:**
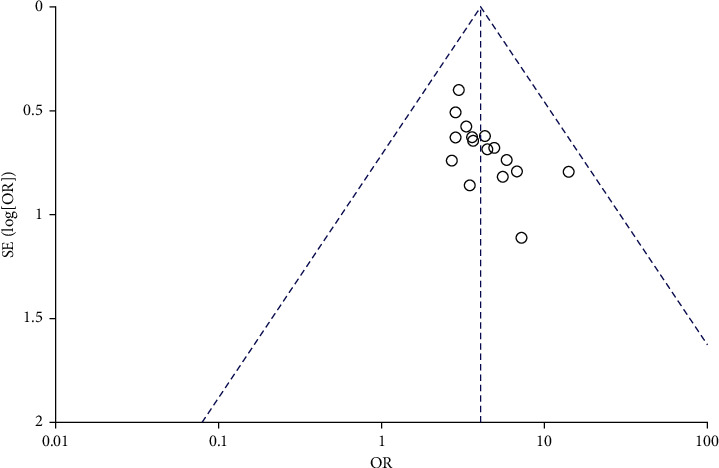
Funnel plots of clinical efficacy.

The clinical efficacy of honghua injection combined with western medicine in the treatment of coronary heart disease angina pectoris was analyzed by publication bias, and an inverted funnel chart was made. The analysis showed that the bilateral symmetry of the funnel chart was acceptable, indicating that the publication bias was unknown (Figure 9).

## 4. Discussion

Inflammation is one of the main causes of coronary plaque. Myocardial infarction (heart attack) is a common manifestation of CAD. CAD can lead to cardiovascular complications to a large extent. The role of oxidative stress or inflammation predisposes atherosclerotic plaques to rupture, leading to CV events (CAD), so inflammation is likely to be a driver of CAD and atherosclerotic plaque development [33, 34].

The types of coronary heart disease included in this meta-analysis include unstable angina, acute myocardial infarction, and coronary heart disease myocardial ischemia. The control group used conventional western medicine for the clinical treatment of coronary heart disease angina pectoris, such as statins, aspirin, enteric-coated tablets, nitrates, etc. The frequency of administration was mostly once a day, up to three times a day. The treatment group was honghua injection plus the control group. Outcome indicators include not only short-term key efficacy indicators but also physical and chemical indicators.

The results of this meta-analysis showed that the efficacy of honghua injection combined with western medicine in the treatment of coronary heart disease angina pectoris is better than that of western medicine alone, which is manifested in significantly improving clinical efficacy, electrocardiogram, and blood lipid indicators. At present, the clinical treatment of coronary heart disease angina pectoris also pays more attention to the combination of Chinese and western medicine, which has the advantages of small toxic side effects and obvious therapeutic effects.

### 4.1. Limitation

(1) According to the scores of the Cochrane Risk Evaluation Form, the quality of the studies included in this study is not high. Most of the random methods only describe “random” without specifying the specific random allocation method; and all studies did not mention whether blinding and allocation concealment were used. (2) Since only a few of the included literature mentioned adverse reactions, the safety evaluation of honghua injection needs to be studied. (3) The number of studies on a single outcome indicator is too small (for example, only 4 studies are included), and there is no subgroup analysis for indicators with heterogeneity to explore the source of heterogeneity. (4) The studies involved in this study have not been followed up for long-term.

Future trial design needs to increase the sample size, design strict randomized controlled trials, adopt appropriate randomization methods, and increase the use of blinding and allocation concealment methods in trials; improve the strength of research evidence, objectively report negative results, and ensure the statistics. The authenticity of the data provides a large amount of reliable data support for the treatment of coronary heart disease angina pectoris to better guide clinical practice (Tables 3 and 4).

## Figures and Tables

**Table 1:** Characteristics of included studies.

Included in the study	Sample size		Gender: (male/female)		Average age (years)		Intervention		Course oftreatment	Outcome indicators
	T	C	T	C	T	C	T	C		
Zhao 2019 [12]	42	40	24/18	21/19	63.8 ± 7.5	64.2 ± 7.2	Honghuainjection + C	Simvastatin	28 d	1
Zhang 2018 [13]	98	98	51/47	52/46	62.2 ± 5.8	61.7 ± 6.1	Honghuainjection + C	Basic treatment, including oxygen inhalation and sublingualadministration of nitroglycerin tablets; for severe cases, calciumantagonists to prevent angina pectoris and low molecular weight heparinto strengthen anticoagulation; aspirin tablets to prevent plateletaggregation; treatment with angiotensin converting enzyme inhibitors.Isosorbide mononitrate injection	14 d	1
Cao 2017 [30]	39	39	23/16	24/15	60.4 ± 10.4	60.9 ± 10.9	Honghuainjection + C	Atorvastatin, all patients receive oxygen therapy, bed rest,oral *β*-blockers, aspirin and other drugs	15 d	3, 4, 5, 6
Cao 2016 [31]	60	60	34/26	32/28	62.4 ± 11.6	61.9 ± 12.3	Honghuainjection + C	All are given conventional treatment, the main drugs are nitrates,statins, antiplatelet aggregation drugs and *β*-receptor blockers,of which atorvastatinis used for statins	14 d	3, 4, 5, 6
To 2014 [14]	30	30	—	—	—	—	Honghuainjection + C	Conventional treatment of coronary heart disease	14 d	1, 2
Wang 2014 [15]	30	30	16/14	13/17	53.2 ± 1.4	56.5 ± 1.4	Honghuainjection + C	Aspirin enteric-coated tablets, metoprolol tartrate,nifedipine sustained-release tablets, statins lipid-loweringdrugs. Isosorbide mononitrate injection	15d	1
Liao 2013 [16]	50	50	—	—	—	—	Honghuainjection + C	Aspirin enteric-coated tablets	28 d	1, 3, 4, 5, 6
Lin 2012 [32]	35	35	21/14	20/15	62.7 ± 5.8	62.4 ± 5.9	Honghuainjection + C	Nitrate, lipid-lowering, antiplatelet, beta blocker	14 d, 28 d	3, 4, 5, 6
Wang 2012 [17]	32	32	17/15	19/13	56.4	55.4	Honghuainjection + C	Basic treatment is given, including oxygen inhalation,aspirin to prevent platelet aggregation, nitrate ester drugs to dilatecoronary arteries, etc., and *β*-receptorblockers, angiotensin converting enzyme inhibitors or angiotensin II receptorantagonists can be selected according to the needs of the disease medicine etc	14 d	1, 2
Li 2012 [18]	50	50	34/16	32/18	—	—	Honghuainjection + C	Both groups received conventional treatment, namely oral enteric-coatedaspirin, isosorbide, metoprolol	14 d	1
Li 2012 [19]	32	32	21/11	23/9	—	—	Honghuainjection + C	Aspirin enteric-coated tablets	14 d	1, 2
Xiao 2012 [20]	40	40	22/18	21/19	58.0	42.0	Honghuainjection + C	Routine oral long-acting isolaridine; aspirin; and when necessary,calcium ion antagonists, *β*-receptor blockers and other drug treatments,such as sublingual nitroglycerin when angina pectoris occurs	14 d	1
Su 2011 [21]	50	50	27/23	26/24	67.4	68.2	Honghuainjection + C	Conventional treatment of coronary heart disease	14 d	1, 2
Ai 2011 [29]	48	48	—	—	—	—	Honghuainjection + C	Nifedipine	14 d	2
Fan 2010 [22]	80	80	46/34	44/36	61.8	60.5	Honghuainjection + C	Enteric-coated aspirin, medicinal isosorbidemononitrate, simvastatin capsules	28 d	1, 2
Zhang Jilei 2010 [23]	46	46	24/21	23/22	66.9	68.7	Honghuainjection + C	The conventional treatment of coronary heart disease and anginapectoris includes oxygen inhalation, isosorbide dinitrate tablets,enteric-coated aspirin tablets, etc., and *β*-receptor blockers and angiotensinconverting enzyme inhibitors are selected according to the condition	14 d	1, 2
Zhang Jingru 2010 [24]	41	41	26/15	28/13	59.0	52.0	Honghuainjection + C	Isosorbide dinitrate, betaloc, enteric-coated aspirin and diltiazem	30 d	1, 2
Cheng 2009 [25]	68	40	38/30	24/16	59.1	58.7	Honghuainjection + C	Nitrate (mainly intravenous drip of nitroglycerin)low-dose aspirin; calcium antagonist, beta blocker	14 d	1, 2
Du 2009 [26]	30	30	—	—	—	—	Honghuainjection + C	Conventional treatment group. Treatmentincludes aspirin, nitrates, *β*-receptor blockers, calcium antagonists, etc.	10 d	1
You 2002 [27]	36	30	22/14	18/12	58.3 ± 8.1	56.4 ± 7.6	Honghuainjection + C	Routine oral long-acting isolaridine; aspirin; and when necessary,calcium ion antagonists, *β*-receptor blockers and other drugtreatments, such as sublingual nitroglycerin when angina pectoris occurs	14 d	1, 2
Zhang 2002 [28]	32	24	—	20/4	—	—	Honghuainjection + C	Conventional treatment of coronary heart disease	15 d	1, 2

Note: 1. Clinical efficacy; 2. ECG efficacy; 3. Total cholesterol (TC); 4. Triacylglycerol (TG); 5. High-density lipoprotein cholesterol (HDL- C); 6. Low-density lipoprotein cholesterol (LDL-C). T: treatment group; C: control group.

**Table 2:** Adverse reactions.

Included in the study	Sample size		Number of adverse reactions		Adverse reactions
	T	C	T	C	
Wang 2012 [17]	32	32	3	0	Mild bloating
Zhang Jilei 2010 [23]	46	46	0	3	1 case had head bloating, 2 cases felt discomfort in the gastric cavity
You 2002 [27]	36	30	1	0	Mild phlebitis, disappears by itself soon after needle removal
Zhang 2002 [28]	32	24	—	—	1 patient had dizziness and skin rash, symptomatictreatment did not affect the curative effect
Zhao 2019 [12]	42	40	8	14	In the observation group, there were 2 cases of facial flushing,2 cases of nausea and vomiting, 1 case of headache,1 case of arrhythmia, 1 case of recurrent myocardial infarction,1 case of post-infarction angina pectoris; controlgroup had 3 cases of nausea and vomiting, 1 case of diarrhea,and 3 cases of heart rhythm disorders, 3 cases of recurrentmyocardial infarction, 2 cases of post-infarction angina,and 2 cases died
Total	188	172	12	17	

**Table 3:** Kappa statistics.

List	Consistency
Included studies (year of first author's name)	√
Sample size (T/C)	√
Gender (male/female) (T/C)	√
Average age (years) (T/C)	√
Interventions (T/C)	√
Course of treatment (T/C)	√
Outcome indicators(T/C)	√

**Table 4:** Grade score.

Outcome indicators	Risk of bias	Inconsistency	Indirect	Imprecision	Publication bias	Evidence level
Clinical efficacy	Severe	Not severe	Not severe	Not severe	Possible	Low
EKG efficacy	Severe	Not severe	Not severe	Not severe	Possible	Low
TC	Severe	Not severe	Not severe	Not severe	Possible	Low
TG	Severe	Not severe	Not severe	Not severe	Possible	Low
HDL-C	Severe	Not severe	Not severe	Not severe	Possible	Low
LDL-C	Severe	Not severe	Not severe	Not severe	Possible	Low
Adverse reactions	Severe	Not severe	Not severe	Not severe	Possible	Low

## Data Availability

All the data generated or analyzed during this study are included in this published article.

## Abbreviations

TC: Total cholesterol
TG: Triacylglycerol
HDL-C: High-density lipoprotein-cholesterol
LDL-C: Low-density lipoprotein-cholesterol
MD: Mean difference
OR: Odds ratio
RCT: Randomized controlled trial.

## Appendix

### GRADE

The GRADE grading grades the quality of evidence for outcome indicators through five downgrading factors: study limitation, inconsistency, immediacy, imprecision, and publication bias. The quality of evidence for trials (RCTs) was rated as high by default, down 1 to intermediate, down 2 to low, and down 3 to very low.

### Search Strategies

((((((CAD[Title/Abstract]) OR (CHD[Title/Abstract])) OR (angina pectoris[Title/Abstract])) OR (coronary syndrome[Title/Abstract])) OR (“Coronary Artery Disease”[Mesh])) AND (((Honghua injection[Title/Abstract]) OR (Hong-hua injection[Title/Abstract])) OR (Safflower injection[Title/Abstract]))) AND (((randomized controlled[Title/Abstract]) OR (random[Title/Abstract])) OR (RCT[Title/Abstract])).
